# Active surveillance in renal transplant patients with prostate cancer: a multicentre analysis

**DOI:** 10.1007/s00345-023-04294-2

**Published:** 2023-01-30

**Authors:** Timo F. W. Soeterik, Roderick C. N. van den Bergh, Harm H. E. van Melick, Hans Kelder, Federica Peretti, Charles Dariane, Marc-Olivier Timsit, Julien Branchereau, Benoit Mesnard, Derya Tilki, Jonathon Olsburgh, Meghana Kulkarni, Veeru Kasivisvanathan, Alberto Breda, Luigi Biancone, Paolo Gontero, Giorgio Gandaglia, Giancarlo Marra, on behalf of the Young Academic Urologists Prostate Cancer Working Party (YAU-PCa WP), Oussama Hedli, Cedric Lebacle, Jacques Irani, Oscar Rodriguez-Faba, Paola Todeschini, Constance Thibault, Josep M. Gaya, Gaetano Lamanna, Antonio Secchi

**Affiliations:** 1 Department of Urology, St. Antonius Hospital, Nieuwegein, Utrecht, The Netherlands; 2 Department of Surgical Sciences, University of Turin and Città della Salute e della Scienza, Turin, Italy; 3 Department of Urology, Hôpital Européen Georges-Pompidou, University of Paris, Paris, France; 4 Department of Urology, CHU Nantes, Nantes, France; 5 Martini-Klinik Prostate Cancer Center, University Hospital Hamburg-Eppendorf, Hamburg, Germany; 6 Department of Urology, Guy’s Hospital, London, UK; 7 Department of Urology, University College London Hospitals NHS Foundation Trust, London, UK; 8 Department of Urology, Fundacio Puigvert, Barcelona, Spain; 9 Department of Urology, San Raffaele Hospital, Milan, Italy; 10 Department of Nephrology, University of Turin and Città Della Salute E Della Scienza, Turin, Italy; 11 Department of Urology, Institut Mutualiste Montsouris and Université Paris Descartes, Paris, France; 12 Department of Urology, Hôpital Tenon, Paris, France; 13 Department of Urology, University Hospital Hamburg-Eppendorf, Hamburg, Germany; 14 Department of Urology, Koc University Hospital, Istanbul, Turkey

**Keywords:** Prostate cancer, Renal transplantation, Active surveillance

## Abstract

**Introduction:**

Due to medical improvements leading to increased life expectancy after renal transplantation and widened eligibility criteria allowing older patients to be transplanted, incidence of (low-risk) prostate cancer (PCa) is increasing among renal transplant recipients (RTR). It remains to be established whether active surveillance (AS) for PCa represents a safe treatment option in this setting. Therefore, we aim to compare AS discontinuation and oncological outcomes of AS for PCa of RTR vs. non-transplant patients.

**Methods:**

Multicentre study including RTR diagnosed with PCa between 2008 and 2018 in whom AS was initiated. A subgroup of non-RTR from the St. Antonius hospital AS cohort was used as a control group. Comparison of RTR vs. non-RTR was performed by 2:1 propensity score matched survival analysis. Outcome measures included tumour progression-free survival, treatment-free survival, metastasis rates, biochemical recurrence rates and overall survival. Patients were matched based on age, year of diagnosis, PSA, biopsy ISUP grade group, relative number of positive biopsy cores and clinical stage.

**Results:**

A total of 628 patients under AS were evaluated, including 17 RTRs and 611 non-RTRs. A total of 13 RTR cases were matched with 24 non-RTR cases. Median overall follow-up for the RTR and non-RTR matched cases was, respectively, 5.1 (IQR 3.2–8.7) years and 5.7 (IQR 4.8–8.1) years. There were no events of metastasis and biochemical recurrence among matched cases. The matched-pair analysis results in a 1-year and 5-year survival of the RTR and non-RTR patients were, respectively, 100 vs. 92%, and 39 vs. 76% for tumour progression, 100 vs. 91% and 59 vs. 76% for treatment-free survival and, respectively, 100 vs. 100% and 88 vs. 100% for overall survival. No significant differences in tumour progression-free survival (*p* = 0.07) and treatment-free survival were observed (*p* = 0.3). However, there was a significant difference in overall survival comparing both groups (*p* = 0.046).

**Conclusions:**

AS may be carefully considered in RTR with low-risk PCa. In our preliminary analysis, no major differences were present in AS outcomes between RTR and non-RTR. Overall mortality was significantly higher in the RTR subgroup.

**Supplementary Information:**

The online version contains supplementary material available at 10.1007/s00345-023-04294-2.

## Introduction

Current developments in renal transplantation have led to wider eligibility criteria, as well as longer life expectancy of renal transplant recipients (RTR). Inherent to the increasing age in this population, RTR are increasingly at risk of developing other non-transplant but age-related morbidities, including prostate cancer (PCa) [1]. To decide on the most optimal treatment of PCa in the RTR population, data regarding the outcomes of PCa treatment are crucial. A recent systematic review evaluating 41 studies including a total of 319 RTR diagnosed with PCa showed that most patients with localised PCa are treated with RP (82%), compared to EBRT (12%) or brachytherapy (6%). Oncological outcomes were comparable to the non-RTR PCa populations, with reported 5-year cancer-specific survival of 97.5% for RP, 87.5% for EBRT and 94.4% for brachytherapy, respectively. [1] In another systematic review, similar conclusions were detailed [2].

As we previously reported, there is lack of good-quality evidence regarding the outcomes of PCa in RTR, and number of studies reporting clinical outcomes is limited [2]. For active surveillance (AS) in particular, no outcomes have been reported in RTR [3]. Due to this lack of evidence and the theoretical higher risk of disease progression due to immunosuppression, AS is not considered the preferred treatment in newly diagnosed low-risk PCa and RTRs with low-risk PCa may be at risk of overtreatment. Furthermore, mortality in RTRs is relatively high and increased compared to non-RTRs [4]. Therefore, studies evaluating the outcomes of AS for PCa in RTR are urgently needed.

In this multicentre international study, we evaluate the outcomes of AS in RTR and compared them with those of AS performed in a standard non-RTR cohort.

## Patients and methods

### Study population

#### Renal transplantation AS cohort

We retrospectively collected data of men being diagnosed with histologically documented PCa after kidney transplant at seven European tertiary referral centres between 2001 and 2019.

All patients performed staging according to the European Association of Urology (EAU) guidelines (axial abdominal imaging—multiparametric magnetic resonance imaging (mpMRI) and/or computer tomography (CT) scan and bone scan). Two physicians independently performed data quality review (G.M., and T.F.W.S). Centres were re-contacted for data revision in case of uncertainty or missing information.

For all centres, principles of the Prostate Cancer International Active Surveillance (PRIAS) study were followed [5]. Variations were allowed depending on the treating physician and national and local guidelines.

#### Non-transplantation control AS cohort

The RTR were matched with non-transplant patients from the AS cohort of St. Antonius Hospital Nieuwegein, who were diagnosed with PCa from 2008 to 2018. Patients were included following the eligibility criteria of to the PRIAS study. Follow-up and discontinuation criteria were also in agreement with the PRIAS study protocol [5]. Compliance with the PRIAS study protocol, as well as the AS outcomes in this cohort, have been reported previously. [6, 7]

### Primary and secondary outcomes

The primary outcome assessed in this study was rate of tumour progression during AS. Secondary outcomes included active treatment rates, biochemical recurrence rates, metastasis rates and overall and PCa-specific survival.

### Statistical analysis

Patients were matched through a propensity score 2:1 matched cox regression analysis. Patients were matched based on age, year of PCa diagnosis, PSA, biopsy International Society of Urological Pathology (ISUP) grade group, relative number of positive biopsy cores and clinical stage assessed by digital rectal examination. Comparative analysis of continuous values was done using the Student’s *T* test or Mann–Whitney *U* test where appropriate. Comparison of categorical values was done using Fisher’s exact test. Kaplan–Meier curves with log rank test and Cox regression analysis were used to establish progression-free survival and to compare time-dependent endpoints. Statistical analysis was performed using IBM SPSS Statistics for Windows, version 26 (IBM Corp., Armonk, N.Y., USA) and R v3.6.3. (R Project for Statistical Computing, www.r-project.org)

## Results

### Patient cohorts

A total of 628 patients were evaluated, including 17 RTRs and 611 non-transplant PCa patients in the control group. The baseline characteristics of both cohorts are presented in Table 1. As shown, substantial differences comparing RTRs and the non-transplant group regarding PSA (5.5 vs. 6.9, *p* = 0.050) and clinical T stage (e.g. cT2 41 vs. 17%, *p* = 0.001) could be observed in the non-matched population. A total of 13 RTR were matched to 24 non-RTR cases. Of these, 11 RTR could be matched with, respectively, two independent non-RTR cases, whereas two RTR could be matched with, respectively, one non-RTR case. After propensity score matching of a total of 13 RTR to 24 non-transplant PCa patients, no statistically significant differences in baseline characteristics were observed (Table 1).

**Table 1:** Baseline characteristics of the renal transplant and non-transplant cohort

	Unmatched			Matched		
	Renal transplant cohort *N* (%)	Non-transplant cohort *N* (%)	*P* value	Renal transplant cohort *N* (%)	Non-transplant cohort *N* (%)	*P* value
No. of patients	17	611		13	24	
Age, years (median, IQR)	65.0 (59–72)	67 (63–72)	0.225	68 (59–72)	70 (62–72)	0.479
PSA (ng/ml) (median, IQR)	5.5 (2.0–7.8)	6.9 (5.1–9.5)	0.050	6.0 (4.2–8.0)	5.4 (3.5–8.0)	0.582
Percentage of positive cores at diagnosis	17	20	0.319	0.17	0.10	0.494
Clinical T stage						
T1a/b T1c T2 T3	5 (29) 4 (24) 7 (41) 1 (6)	65 (11) 428 (70) 105 (17) 13 (2)	0.001	4 (31) 3 (23) 6 (46) 0 (0)	7 (29) 6 (25) 11 (46) 0 (0)	0.965
Biopsy ISUP grade						
Group 1 Group 2 Group 3 Group 4 Group 5	15 (88) 2 (12) 0 (0) 0 (0) 0 (0)	558 (91) 45 (7) 7 (1) 1 (0) 0 (0)	0.881	12 (92) 1 (8) 0 (0) 0 (0) 0 (0)	23 (96) 1 (4) 0 (0) 0 (0) 0 (0)	1.0
Year of diagnosis						
2008–2013 2014–2018	9 (53) 8 (47)	258 (42) 353 (58)	0.458	7 (54) 6 (46)	12 (50) 12 (50)	1.0

Detailed renal transplantation-related baseline features of the RTR cohort are presented in Table 2. As shown in Table 2, transplantation year ranged from 1977 to 2013. The cause of renal failure was chronic in the majority of cases (97%) and a wide variety of immunosuppression schedules were applied (Table 2).

**Table 2:** Baseline transplant-specific characteristics of the renal transplant recipients at PCa diagnosis

Patient and transplant features	
Transplantation year	
1977–1997 1999–2009 2010–2013	4 (24) 8 (48) 5 (30)
Time between transplantation and PCa diagnosis (years), median, range	120 (48–206)
Age (years), SD	65 (7.3)
ASA score	
1 2 3 Unknown	0 (0) 5 (29) 9 (52) 3 (18)
Other malignancy	
Kidney cancer Skin cancer (non-melanoma) Kaposi’s Sarcoma Thyroid Gland None	2 (12) 2 (12) 1 (6) 1 (6) 11 (65)
Transplant and kidney failure features	
Renal failure	
Acute Chronic	1 (3) 16 (97)
Cause of renal failure	
Chronic glomerulonephritis Autosomal dominant polycystic kidney disease Diabetic nephropathy Nephrosclerosis Urate nephropathy Chronic pyelonephritis Congenital renal dysplasia Others	4 (24) 2 (12) 3 (18) 1 (6) 1 (6) 1 (6) 1 (6) 4 (24)
Previous dialysis	
Yes No	10 (59) 7 (41)
Type of transplant	
Single cadaver Single living donor Unknown	12 (71) 4 (23) 1 (6)
Time from first transplant to PCa in months	
(Median, IQR)	120 (48–206)
Immunosuppression	
Antiproliferative agents CNI Steroids (Prednisone) Antiproliferative agents + mTOR inhibitor Antiproliferative + CNI + Steroids Antiproliferative + CNI Antiproliferative + Steroids CNI + Steroids	2 (12) 2 (12) 0 1 (6) 3 (18) 8 (47) 0 1 (6)

*IQR* interquartile range, *CNI* Calcineurin Inhibitor, *mTOR* mechanistic target of rapamycin

### Discontinuation of AS

Median AS duration was 4.5 years (IQR 2.6–5.9) for RTR and 3.3 years (IQR 1.8–5.4), (*p* = 0.223) for the non-transplant cohort, respectively. During study follow-up, AS was discontinued in, respectively, 13 out of 17 (76%) of the RTRs and 345 out of 611 (56%) of the non-transplant patients. For the RTR subgroup, respectively, 8 out of 17 (47%) patients discontinued AS due to tumour progression versus 205 out of 611 (34%) for non-transplant patients. A detailed description of the reasons for discontinuation of AS is presented in Table 3.

**Table 3:** Reasons for discontinuation of active surveillance in both transplant and non-transplant patients

		Transplant	Non-transplant
Reason	Category	*N* (%)	*N* (%)
Oncological (tumour progression)	PSA rise	4 (31)	42 (12)
	Biopsy upgrading	3 (23)	39 (11)
	Others	0 (0)	21 (6)
	Combination of two or more	1 (8)	103 (30)
	Total	8 (62)	205 (59)
Non-oncological	Competing disease	2 (15)	71 (21)
	Patient anxiety	0 (0)	13 (4)
	Other	0 (0)	2 (1)
	Lost to follow-up	1 (8)	46 (13)
	Death	2 (15)	8 (2)
	Total	5 (38)	140 (41)
Overall		13 (100)	345 (100)

### Choice of deferred treatment

In the RTR group, 35% of the patients underwent deferred active treatment versus 33% of the patients in the non-transplant group. Mean time from PCa diagnosis to active treatment was, respectively, 4.5 ± 2.5 years for RTR and 3.9 ± 2.6 years for the non-transplant group. Among six RTRs undergoing active treatment, three patients (50%) underwent radical prostatectomy (RP), one patient (17%) underwent brachytherapy, one patient (17%) underwent androgen deprivation therapy (ADT) and one patient (17%) underwent EBRT plus ADT. For the 199 patients who underwent active treatment in the non-transplant group, respectively, 67 patients (34%) were treated with RP, 81 patients (41%) with EBRT, 34 patients (17%) with brachytherapy, five patients (3%) with ADT, nine patients (5%) with EBRT + ADT and three patients (2%) with focal therapy (*p* = 0.120) (Supplemental section, Table S1).

### Outcomes of deferred surgery

The outcomes of deferred radical prostatectomy are presented in Table S1, Supplemental section. As shown, deferred surgery in RTRs resulted in pT2 tumours in 3/3 (100%) of patients, whereas for the non-transplant cohort, pT2, pT3a and pT3b were present in, respectively, 50 (78%), 9 (14%) and 5 (8%) out of 67 patients. In the RTR and non-transplant subgroups, respectively, 1 out of 3 (33%) and 9 out of 64 (13%) underwent concomitant pelvic lymph node dissection, all being pN0.

### Overall and PCa-specific mortality

Median total follow-up time were, respectively, 5.6 (3.2–8.8) years for RTR and 5.0 (IQR 3.2–7.7) years for non-RTR. During study follow-up, 35% of RTR died compared with 8% in of non-RTR (*p* = 0.002). In both groups, no PCa-specific mortality was observed.

### Propensity score matched analysis of AS outcomes

**Fig. 1:**
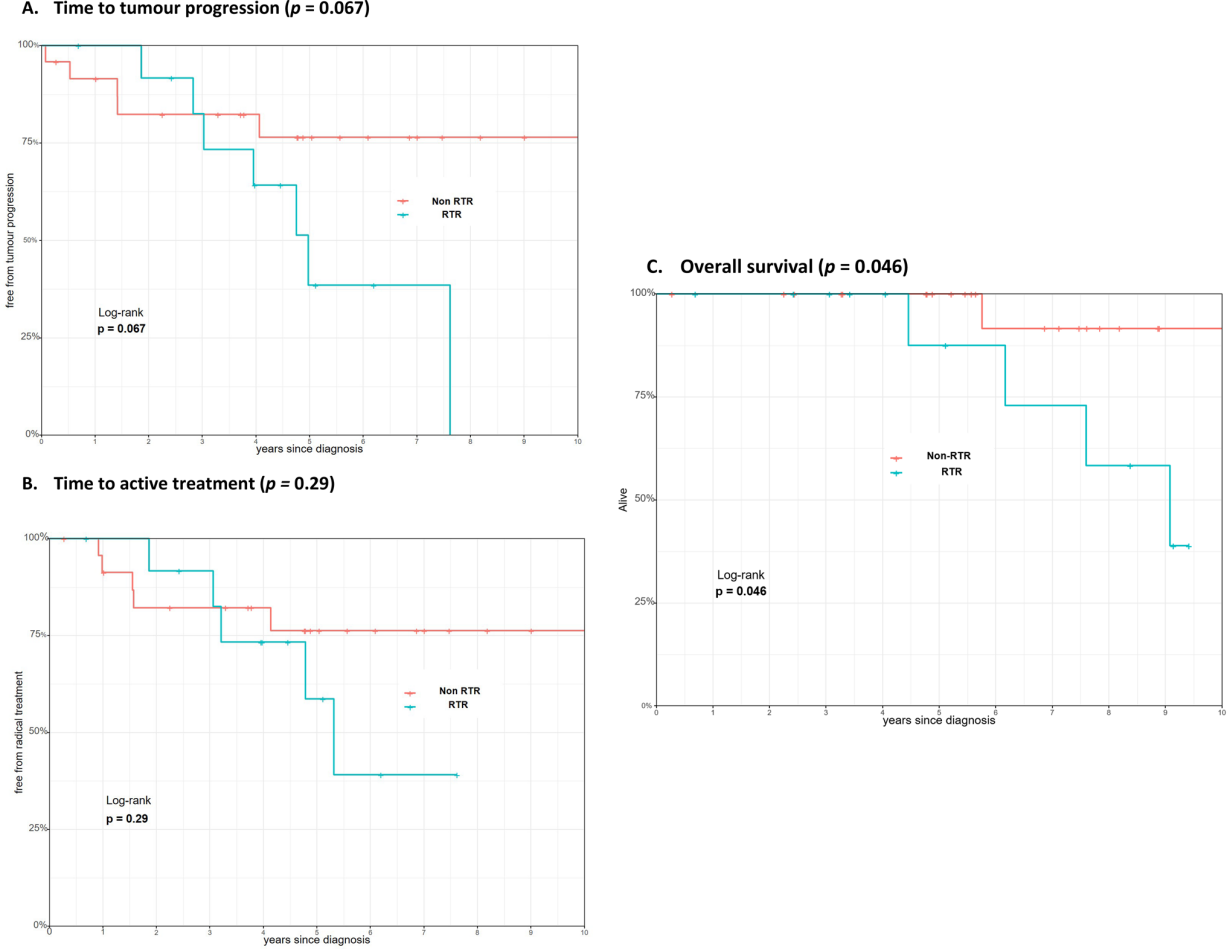
Survival analysis. **A** Time to tumour progression (*p* = 0.067). **B** Time to active treatment (*p* = 0.29). **C** Overall survival (*p* = 0.046)

Median overall follow-up for the RTR and non-RTR matched cases was, respectively, 5.1 (IQR 3.2–8.7) years and 5.7 (IQR 4.8–8.1) years. Among the patients included in the propensity matched analysis (both RTRs and non-transplant patients), no metastasis, biochemical recurrence and PCa-specific mortality were observed during follow-up. The matched-pair analysis resulted in a 1-year and 5-year survival of the RTR and non-RTR patients that were respectively 100 vs. 92%, and 39 vs. 76% for tumour progression, 100 vs. 91% and 59 vs. 76% for treatment-free survival and, respectively, 100 vs. 100% and 88 vs. 100% for overall survival. Kaplan–Meier analysis resulted in no significant differences in tumour progression-free survival (*p* = 0.067). Also, no significant differences in treatment-free survival were observed (*p* = 0.29). However, there was a significant difference in overall survival comparing both groups (*p* = 0.046) (Fig. 1).

## Discussion

To our best knowledge, this is the first study detailing outcomes of AS in RTR and comparing them with non-transplant PCa patients. The most important insight provided by this study is that AS seems safe for RTR. Overall and on propensity score-matched analysis, RTR patients had a significantly lower overall survival compared with the non-RTR matched cases. No significant differences regarding tumour progression during AS and initiation of active treatment were observed.

At present, active treatment is frequently advised in RTR who are diagnosed with low-risk PCa, of which radical prostatectomy remains the most frequently chosen treatment strategy [3]. Currently, no major increase in complications has been detailed overall in RTR when surgery for PCa is performed in tertiary referral centres [2]. Nonetheless, performing RARP may be more challenging compared to non-RTR and requires technical modifications to the standard technique [8]. Therefore, selection of RTR for surgical treatment of PCa should be done carefully.

Moreover, the choice of active treatment in RTR when diagnosed with low-risk prostate cancer may be merely on the basis of fear of poor outcomes due to concomitant immunosuppressive therapy combined with the lack of evidence regarding AS outcomes in this subgroup [3]. The increased risk of unfavourable PCa outcomes in RTRs, related to immunosuppression, was proposed by Kleinclauss et al., who reported that RTR are at risk for early occurrence and for locally advanced PCa, especially when treated with a calcineurin Inhibitor and azathioprine immunosuppressive therapy. [9]

In this study, including 17 RTRs treated with either CNI, antiproliferative agents, or a combination of both, no significant differences in rates of tumour progression between RTRs and non-transplant patients were observed. To our best knowledge, this is the largest series of RTR patients treated with AS for prostate cancer described in the literature. Therefore, the information that can be extracted from this cohort is crucial to improve the understanding of prostate cancer prognosis in this specific population.

For instance, an important observation is that no PCa metastasis and no BCR cases among RTRs were detailed, arguing against a greater likelihood of (immunosuppression-related) tumour progression in RTRs, which was previously reported. [9] Our inconsistent finding could be explained by major differences in patient populations, as the cohort described by Kleinclauss et al. consisted of patients with follow-up from 2004 to 2005. In that time period, there were inherent differences in patient selection and follow-up for AS [9]. The cohort described in our study includes more recently diagnosed PCa patients (2008 to 2018). In this period, AS protocols have been standardised and evidence from numerous AS studies emerged, providing information to further improve the safety of AS. [10]

In the propensity score-matched analysis, we observed significantly worse overall survival for the RTR group compared with the non-RTR group. This finding may encourage the choice for AS in RTRs who are diagnosed with low-risk PCa. As there is a relatively high likelihood patients will die from competing disease, subjecting them to active treatment and thus the treatment-related morbidity may not be the preferred option. However, it should be noted that the patients with PCa included in this study, selected for AS, might constitute of a subgroup of RTR patients with worse prognosis. For example, when comparing mortality rates with those reported in a large series of kidney transplant recipients, a 10-year mortality of 22.1% was reported [4]. In our study, 5-year mortality in the RTR group was, respectively, 35%. This may indicate a selection of a specific subgroup with an overall worse prognosis compared with the general RTR population.

The overall greater likelihood of dying from non-disease-related causes for selected PCa RTRs with expectant management is supported by an analysis of SEER-Medicare data by Liauw et al., including the comparison of PCa treatment outcomes of 620 patients (including 320 patients with transplant before diagnosis) with 3100 non-transplant patients [11]. Choice of treatment was, respectively, prostatectomy in 16% of patients, radiation therapy (with or without ADT) in 43%, hormonal therapy in 18% and 23% no active treatment. The authors reported a significant higher overall mortality at ten years for RTRs vs. non-transplant patients (56 vs. 42%, *p* < 0.001) [11].

Altogether, the relatively higher likelihood of dying from competing diseases other than PCa among RTRs, established in the present and prior study, should be considered as another argument that either AS or watchful waiting are suitable treatment options for PCa in appropriately selected RTRs.

Our study is not exempt of limitations. First, it has a small sample size. Nonetheless, to our knowledge, this is the first and largest multi-institutional series detailing outcomes of PCa AS for RTR. Second, there is a lack of universally used and pre-specified AS protocol. Thus, patient selection and adherence may have differed among centres. Third, a large percentage of the patient population was diagnosed with PCa before mpMRI-based diagnosis had become standard of care, which limits the generalisability of the AS cohort. Finally, since AS is not common in RTR, selection bias may have occurred as shown by our relatively high mortality rates in the RTRs group. Hence, no strict recommendations can be extracted from our work. Whilst it seems no major contra-indication exists to perform AS in RTR based on our results, prospective, and favourably randomised controlled trials, should be awaited to further confirm the safety of AS for low-risk PCa in RTR.

## Conclusions

AS yields good outcomes with no cases of BCR and/or metastatic progression and/or cancer-related deaths in selected RTRs patients affected by localised PCa. No major significant differences were observed regarding AS outcomes compared to non-transplant PCa patients except for overall mortality, being worse in RTRs patients as expected. AS does not seem an unsafe option for low-risk PCa in RTR and should be carefully considered in appropriately selected patients.

## Supplementary Information

Below is the link to the electronic supplementary material.

Supplementary file1 (DOCX 15 KB)

## Data Availability

The datasets generated during and/or analysed during the current study are available from the corresponding author on reasonable request.

## References

[1] Hevia V, Boissier R, Rodríguez-Faba Ó (2018). Management of localised prostate cancer in kidney transplant patients: a systematic review from the EAU guidelines on renal transplantation panel. Eur Urol Focus.

[2] Marra G, Dalmasso E, Agnello M (2018). Prostate cancer treatment in renal transplant recipients: a systematic review. BJU Int.

[3] Aminsharifi A, Simon R, Polascik TJ (2019). Evaluation and active treatment versus active surveillance of localized prostate cancer in renal transplant patients in the era of low and very low risk prostate cancer. J Urol.

[4] Awan AA, Niu J, Pan JS (2018). Trends in the causes of death among kidney transplant recipients in the United States (1996–2014). Am J Nephrol.

[5] van den Bergh RCN, Roemeling S, Roobol MJ, Roobol W, Schröder FH, Bangma CH (2007). Prospective validation of active surveillance in prostate cancer: The PRIAS Study. Eur Urol.

[6] Soeterik TFW, van Melick HHE, Dijksman LM, Biesma DH, Witjes JA, van Basten JPA (2018). Active surveillance for prostate cancer in a real-life cohort: comparing outcomes for PRIAS-eligible and PRIAS-ineligible patients. Eur Urol Oncol.

[7] Soeterik TFW, van Melick HHE, Dijksman LM, Biesma DH, Witjes JA, van Basten JPA (2019). Follow-up in active surveillance for prostate cancer: strict protocol adherence remains important for PRIAS-ineligible patients. Eur Urol Oncol.

[8] Marra G, Agnello M, Giordano A (2022). Robotic radical prostatectomy for prostate cancer in renal transplant recipients: results from a multicenter series. Eur Urol.

[9] Kleinclauss F, Gigante M, Neuzillet Y (2008). Prostate cancer in renal transplant recipients. Nephrol Dial Transplant.

[10] Tosoian JJ, Carter HB, Lepor A, Loeb S (2016). Active surveillance for prostate cancer: contemporary state of practice. Nat Rev Urol.

[11] Liauw SL, Ham SA, Das LC (2020). Prostate cancer outcomes following solid-organ transplantation: a seer-medicare analysis. J Natl Cancer Inst.

