# Supplementary material for: Active surveillance in renal transplant patients with prostate cancer: a multicentre analysis
Source: World J Urol. 2023 Jan 30;41(3):725–32. doi: 10.1007/s00345-023-04294-2 (PMC10082698; doi:10.1007/s00345-023-04294-2)
Supplement: Supplementary file 1 — Supplementary file1 (DOCX 15 KB) [file 345_2023_4294_MOESM1_ESM.docx]

**Supplemental section**

**Table S1. Choice of deferred treatment and outcomes of radical prostatectomy**

|  | **Renal transplant cohort**  **N (%)** | **Non-transplant cohort**  **N (%)** | ***p*** |
| --- | --- | --- | --- |
| Active treatment type  Radical prostatectomy  EBRT  Brachytherapy  ADT  EBRT + ADT  Focal therapy  Total | 3 (50)  0 (0)  1 (17)  1 (17)  1 (17)  0 (0)  6 (100) | 67 (34)  81 (41)  34 (34)  5 (3)  9 (5)  3 (2)  199 (100) | 0.120 |
| Radical prostatectomy pathology  ISUP  1  2  3  4  Unknown  pT  2  3a  3b  Unknown  PSM  Positive  Negative  Unknown  pN  0  1  X  Unknown  Total | 1 (33)  2 (67)  0 (0)  0 (0)  0 (0)  3 (100)  0 (0)  0 (0)  0 (0)  0 (0)  3 (100)  0 (0)  1 (33)  0 (0)  2 (67)  0 (0)  3 (100) | 12 (18)  35 (52)  12 (19)  4 (6)  4 (6)  50 (78)  9 (14)  5 (8)  4 (6)  20 (30)  42 (63)  4 (6)  9 (13)  0 (0)  54 (81)  4 (6)  67 (100) | 0.756  0.128  0.542  0.389 |
